# Protein microparticles visualize the contact network and rigidity onset in the gelation of model proteins

**DOI:** 10.1038/s41538-021-00111-5

**Published:** 2021-12-13

**Authors:** Joep Rouwhorst, Carlijn van Baalen, Krassimir Velikov, Mehdi Habibi, Erik van der Linden, Peter Schall

**Affiliations:** 1 Institute of Physics, University of Amsterdam, Science Park 904, 1098 XH Amsterdam, The Netherlands; 2 Physics and Physical Chemistry of Foods, Wageningen University, Bornse Weilanden 9, 6708 WG Wageningen, The Netherlands; 3 Unilever Foods Innovation Centre, Bronland 14, 6708 WH Wageningen, The Netherlands

**Keywords:** Gels and hydrogels, Biological physics

## Abstract

Protein aggregation into gel networks is of immense importance in diverse areas from food science to medical research; however, it remains a grand challenge as the underlying molecular interactions are complex, difficult to access experimentally, and to model computationally. Early stages of gelation often involve protein aggregation into protein clusters that later on aggregate into a gel network. Recently synthesized protein microparticles allow direct control of these early stages of aggregation, decoupling them from the subsequent gelation stages. Here, by following the gelation of protein microparticles directly at the particle scale, we elucidate in detail the emergence of a percolating structure and the onset of rigidity as measured by microrheology. We find that the largest particle cluster, correlation length, and degree of polymerization all diverge with power laws, while the particles bind irreversibly indicating a nonequilibrium percolation process, in agreement with recent results on weakly attractive colloids. Concomitantly, the elastic modulus increases in a power-law fashion as determined by microrheology. These results give a consistent microscopic picture of the emergence of rigidity in a nonequilibrium percolation process that likely underlies the gelation in many more systems such as proteins, and other strongly interacting structures originating from (bio)molecules.

## Introduction

Protein aggregation is of immense importance in biology, medical research, and food and dairy products^1–12^. It is related to many diseases, including aging-related neurodegeneration and systemic amyloidosis^1,2^: cells avoid accumulating potentially toxic aggregates by suppression of aggregate formation and degradation of misfolded proteins. Understanding protein aggregation is also important for sustainably producing foods^13,14^; as plant proteins are becoming of increasing importance as a sustainable replacement for animal proteins, understanding and designing the structure and corresponding texture of aggregated proteins and protein mixtures is crucial.

Yet, protein interactions are complex and notoriously difficult to model. Protein aggregation is usually induced by a change in the pH, ionic strength, protein concentration, or temperature. Heating induces denaturation of the proteins leading to enlarged exposure of hydrophobic and reactive groups such as thiol groups that cause sticking, while lowering the pH towards the proteins’ isoelectric point eliminates their electrostatic stabilization causing aggregation. While ab initio calculations can predict conformations and interactions of single proteins, the consequent aggregation remains computationally inaccessible, and its modeling has to rely on an effective sticky sphere or patchy particle models^15^.

Experimental insight into protein aggregation is equally challenging, relying mostly on scattering techniques such as light and small-angle x-ray or neutron scattering that yield merely average values of microscopic quantities such as the cluster size^9–11,16–18^, or on rheology providing mechanical signatures of aggregation and gelation^5,7,8^. This makes an insight into the microscopic aggregation mechanism to test the applicability of theories of aggregation and gelation to proteins prohibitively difficult. Protein aggregation typically proceeds via several steps: preaggregation, in which heat-denatured monomers form oligomers and larger primary aggregates, followed by the association of the primary aggregates into self-similar structures that gel at sufficient particle concentration. While usually these steps occur in parallel, making it difficult to disentangle them, cold-set gelation decouples the heat-induced activation from the subsequent gelation step, enabling insight into the later stages of protein aggregation alone. Still, linear chains or fibrils often form initially, and the structure and size of the primary aggregates vary significantly^4^. We, therefore, use cold-set gelation of well-controlled spherical protein aggregates (protein microparticles)^12,19^, serving as models for the primary aggregates. Using these micron-sized protein particles, we provide direct images of the protein gelation process in three dimensions, and we follow the concomitant evolution of rigidity of the growing gel network by microrheology.

The microscopic insight allows us to reveal a new picture behind gelation, which is a ubiquitous phenomenon in many different systems with short- and medium-range attraction, including polymers^20^, proteins^21,22^, and colloids^23–27^, but for which, despite extensive work, no general theory exists: depending on the attraction magnitude, range, and particle density, various scenarios have been proposed to lead to structural arrest and rigidity. At high particle attraction, particles stick as soon as they collide, leading to open, ramified structures at low particle density^28–33^. At low particle attraction, where the system is closer to equilibrium, more compact structures form. Gelation may then occur by spinodal decomposition^23,27^, a thermodynamic instability, in which phase separation into high- and low-density phases occurs spontaneously, followed by the glass-like arrest of the dense phase, leading to structural rigidity^27,34^.

Other mechanisms to reach the arrested state have been proposed, and various interpretations of the equilibrium phase diagrams have been suggested, including equilibrium percolation^35^ and the onset of rigidity percolation^36^.

For proteins, interactions and structures are complex, leading to a multitude of aggregate morphologies and aggregation pathways^4,37^. Among those, globular and weakly denatured proteins have been observed to aggregate into fractal structures with fractal dimensions between 1.7 and 2.3^10,11^, similar to colloidal particles, and similarities in their stability and aggregation have been discussed^9,12,15^. Close to their isoelectric point where the stabilizing electrostatic repulsion ceases, interaction energies are typically large, of the order of several tens of *k*_B_*T*, the thermal energy, and descriptions based on equilibrium phase diagrams may no longer apply as particle attachments become increasingly irreversible and aggregates evolve increasingly slowly over time. Furthermore, due to the complex interplay of electrostatics and pH-dependent dissociation of surface groups, their interaction can change between isolated and crowded environments, making the mechanism of aggregation and gelation prohibitively difficult to model.

We directly follow the cold-set gelation of protein microparticles induced by a change of pH, revealing the cluster growth and evolution of the contact network of nearest neighbors at the particle scale. By tracking the individual particles in the clusters, we follow how the propagating aggregates lead to the emergence of rigidity of the network. The results uncover a nonequilibrium percolation process: particle clusters grow and approach gelation with power-law critical exponents of 1.6 as predicted for three-dimensional percolation models, while at gelation, cluster sizes exhibit a power-law distribution. At the same time, the direct observations show that the particle bonds become clearly irreversible, attesting to the nonequilibrium nature of the process. The results indicate that the network rigidity is governed by bond-bending interactions, while the emergence of the network is governed by a nonequilibrium continuous phase transition process.

## Results

### Rheology

**Fig. 1:**
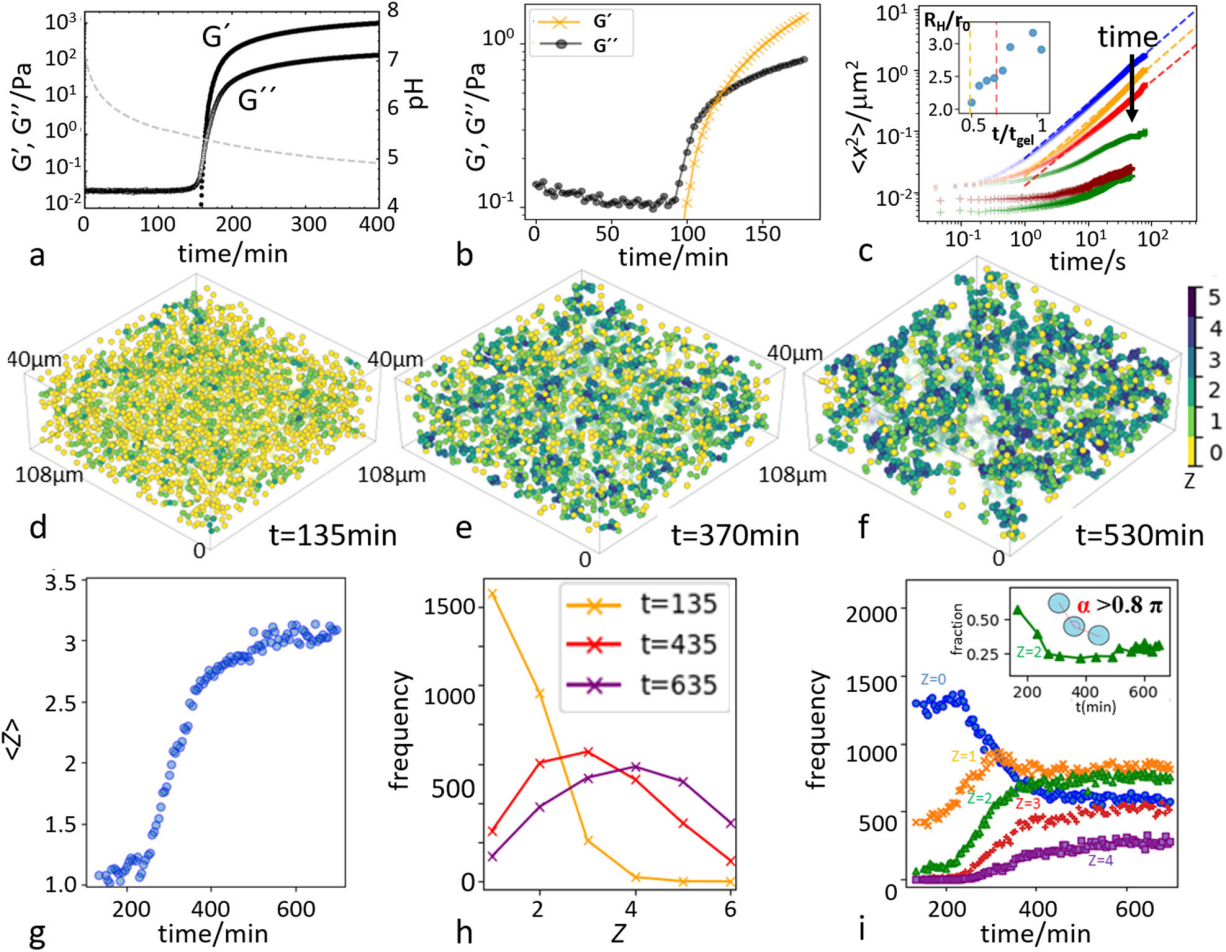
Observation of gelation in protein microparticles. **a**, **b** Storage and loss moduli, ${G}^{\prime}$ and *G″,* show the onset of gelation by a rapid increase of ${G}^{\prime}$ for protein (**a**) and protein microparticle suspension (**b**). **c** Mean-square displacement (MSD) of protein microparticles at various stages of aggregation (upper curves), and MSD corrected by cluster center of mass motion (lower curves). The decrease of the former with time demonstrates the growth of the clusters, while the low plateau of the latter shows the immobility of particles within the clusters. Inset: time evolution of the average hydrodynamic radius of clusters as determined from the MSD curves as a function of aggregation time. **d**–**f** Three-dimensional reconstructions of aggregating protein microparticles after 135 (**a**), 370 (**b**), and 530 min (**c**). Particle color indicates local coordination number, i.e., number of bonded neighbors, *Z*. **g** Average coordination number as a function time. **h** Coordination number distributions after 135, 435, and 635 min. **i** Evolution of low coordination numbers. Decrease of *Z* = 0 and increase of *Z* ≥ 2 particles indicate that single particles connect into clusters. Inset: fraction of double-bonded particles (*Z* = 2) with a bond angle close to *π* indicating ubiquity of single-particle strands.

Rheology results of the acidifying protein suspensions, both the regular proteins and the protein microparticles, are shown in Fig. 1a, b. Both exhibit the characteristic signature of gelation, as shown by the rapid rise of the elastic modulus exceeding the loss modulus, demarcating the onset of a load-bearing structure. The magnitudes of the moduli and time scales of the aggregation are different, which we associate mainly with the different particle sizes and densities, and the different diffusion coefficients of the protein and protein microparticle solutions.

### Three-dimensional particle tracking by confocal microscopy

The advantage of the protein microparticles is that we can follow the gelation process in three dimensions at the particle scale by confocal microscopy. We image individual protein microparticles in a 108 μm by 108 μm by 40 μm volume and determine their positions with an accuracy of 20 nm in the horizontal and 35 nm in the vertical direction using open-source particle locating software^38,39^, see Methods. We identify bonded particles as those separated by less than the inflection point of the pair correlation function, *g*(*r*), and group bonded particles together into clusters. We also follow the dynamics of particles in a horizontal plane roughly ~20 μm into the sample by rapidly acquiring images at a frame rate of 24 s^−1^; these confirm that the grouped particles are indeed bonded with each other.

Three-dimensional reconstructions, shown in Fig. 1d–f, reveal the increasing coordination of the particles, indicated in color, as clusters grow. The coarsening of the structure is directly related to an increasing number of bonded neighbors; this is clearly reflected in the rapid rise of the average number of bonds per particle, 〈*Z*〉, which increases sharply after 300 min, as shown in Fig. 1g. It is also reflected in the bond number distribution, which broadens and shifts to higher *Z*, indicating an increasing number of connected particles, Fig. 1h. The time evolution of specific connectivity is shown in Fig. 1i. The drop in the number of unconnected (*Z* = 0) and a concomitant rise in the number of multiply connected particles (*Z* ≥ 2) indicates the increasing number of well-connected particles until after ~500 min, these numbers saturate and the system reaches a steady state. We notice a considerable fraction of thin, single-particle strands (see inset), different from the thick branches typically associated with the arrested phase-separation mechanism of gelation^40,41^, and from our own work on gelation in weakly attractive colloidal systems^42^. This is indicated by a considerable fraction of particles with a bond angle close to *π*, indicating thin, directed strands, which we associate with the strong protein–protein interactions of the order of 30*k*_B_*T* at the isoelectric point (see Supplementary Note 1 and Supplementary Fig. 3).

### Microrheology

**Fig. 2:**
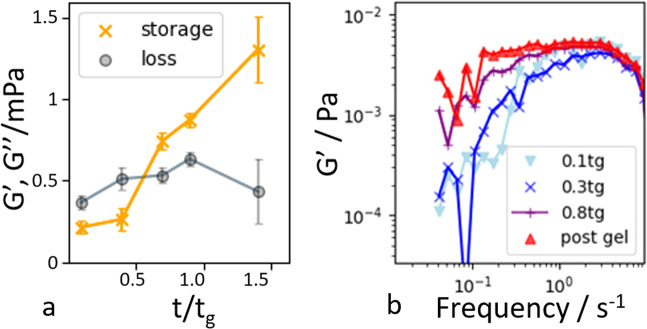
Emergence of microscopic elasticity. **a** Elastic and viscous moduli as a function of normalized time of gelation. Error bars indicate standard deviation. The increase of the elastic modulus above the viscous modulus indicates the emergence of an elastic solid. **b** Elastic modulus versus frequency shows the emergence of a low-frequency plateau from an initial power-law slope.

Concomitantly with the increasing coordination number, the system develops mechanical rigidity as reflected in an emerging elastic modulus. To show this, we cross-correlate the motion of the individual protein particles as a function of their distance to extract the frequency-dependent moduli^43^, see Supplementary Note 2. The resultant moduli shown in Fig. 2a (and Supplementary Fig. 4) indeed reveal the rise of the storage modulus ${G}^{\prime}$ above the loss modulus. The evolution of ${G}^{\prime}$ is similar to that measured by macroscopic rheology (Fig. 1b), and goes parallel with the rise of the average coordination number shown in Fig. 1g. The full frequency-dependent modulus is plotted in Fig. 2b and its full evolution during different stages of aggregation in Supplementary Fig. 4. These figures clearly show the rise of a plateau at low frequency, indicating the emergence of an elastic solid-like material.

### Cluster evolution and critical scaling

**Fig. 3:**
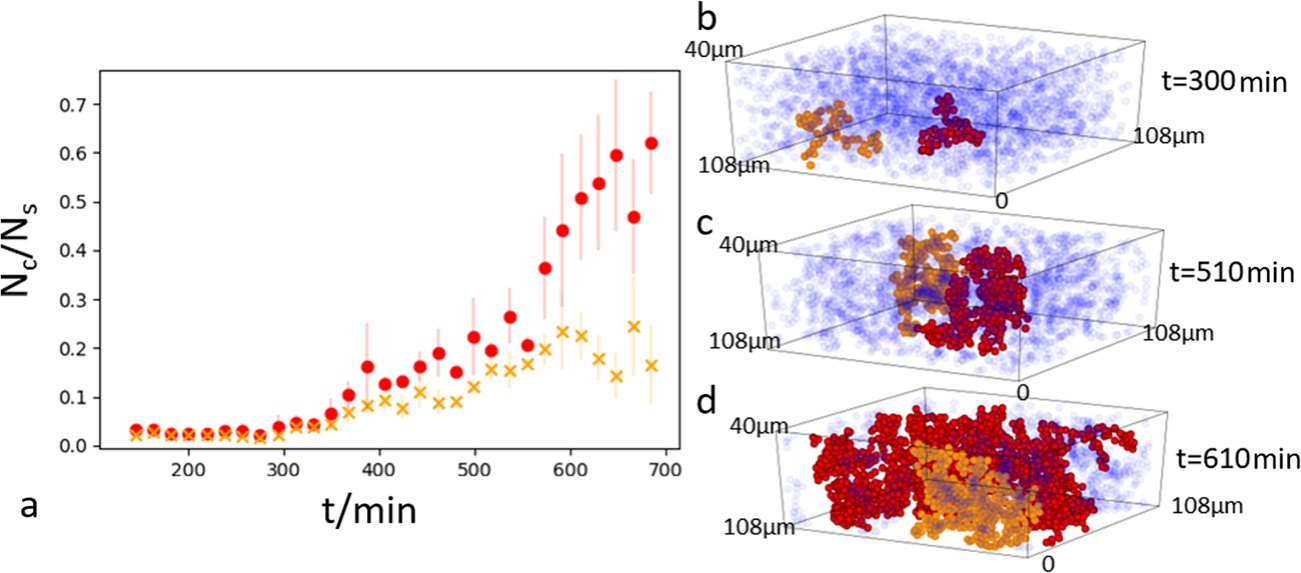
Growth of the largest and second largest cluster. **a** Time evolution of the number of particles in the largest (red) and the second largest cluster (orange). Error bars indicate standard deviation. The rapid increase of the largest cluster and concomitant decline of the second largest cluster indicate the biggest clusters merge and form a space-spanning structure. **b**–**d** Reconstructions of the largest and second largest cluster during the early, intermediate, and late (percolated) stages of the gelation process. Unbonded particles and particles in clusters that are not connected to the two largest clusters are indicated in transparent blue.

The question is how this arrested structure forms. Looking more closely, we find that particles once absorbed by the growing clusters remain fully arrested and immobile, while the clusters themselves exhibit diffusion, see Fig. 1c. Here, we subtracted the displacement of the cluster center of mass from the displacement of each particle; the resulting mean-square displacement of particles within the cluster remains vanishingly small, while the overall mean-square displacement agrees with the expected diffusion of the growing fractal clusters. This breaking of detailed balance in the bonding of particles indicates that the aggregation process is fully irreversible. The resultant evolution of the two largest clusters is shown in Fig. 3, where red data and particles represent the largest cluster and orange the second largest cluster. The largest cluster grows increasingly fast and eventually spans the entire field of view, as shown by the reconstructions on the right.

**Fig. 4:**
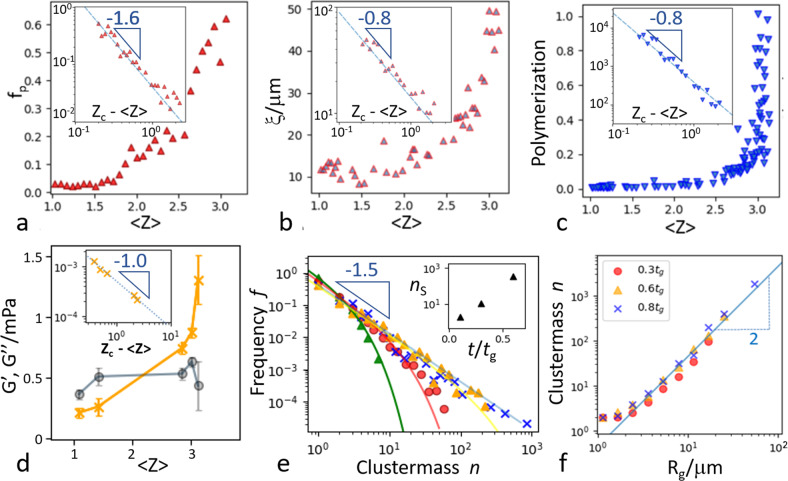
Critical scaling upon approaching gelation. **a** Fraction of particles in the biggest cluster, *f*_p_, as a function of mean coordination number, 〈*Z*〉. Divergence with a power of *α* = 1.6 is observed upon approaching the critical coordination number, *Z*_c_ (inset). **b** Cluster correlation length, *ξ*, versus mean coordination number shows divergence with exponent *ν* = 0.8. **c** Average degree of polymerization versus mean coordination number shows divergence with exponent *γ* = 0.8 (inset). **d** Storage (yellow crosses and line) and loss moduli (gray circles and line) as a function of mean coordination number. Data suggest power-law increase of ${G}^{\prime}$ with exponent ~1 upon approaching the critical coordination number *Z*_c_ (inset). **e** Cluster-mass distributions at 0.1 (green triangles), 0.3 (red dots), 0.6 (orange triangles), and 0.8*t*_*g*_ (blue crosses). The exponential cutoff grows and a power-law with exponent −3/2 emerges upon approaching the gelation time, *t*_g_. Lines indicate the fits $f\propto {n}^{-3/2}\exp (-n/{n}_{S})$. Inset: extracted size cutoff *n*_*S*_ as a function of normalized time. **f** Cluster mass versus radius of gyration of the clusters reveals fractal nature with a fractal dimension *d*_f_ = 2.

Taking the average coordination number 〈*Z*〉 as the order parameter of the gelation process^42^ and plotting the fraction *f*_p_ of particles in the biggest cluster as a function of 〈*Z*〉, we observe a sharp increase upon approaching a critical value *Z*_c_, as shown in Fig. 4a. We find a divergence ${f}_{\rm{p}}\sim {({Z}_{\rm{c}}-\langle Z\rangle )}^{-\alpha }$ with exponent *α* ~ 1.60 ± 0.15 upon approaching the critical connectivity *Z*_c_ = 3.3 ± 0.1. This power-law dependence between cluster size and coordination number was observed by us before for aggregating weakly attractive particles and associated with a new nonequilibrium percolation transition^42^. This picture was supported by a kinetic cluster growth model based on strongly unbalanced particle association and dissociation from the growing clusters. Unlike previous spinodal decomposition and equilibrium percolation scenarios, this new kinetic picture of the gelation process builds genuinely on the breaking of detailed balance in this nonequilibrium process, distinguishing it from the previous scenarios. Concomitantly, the length scale of connected particle clusters diverges. We determine the correlation length of connected particles using ${\xi }^{2}=2{{{\Sigma }}}_{i}{R}_{gi}^{2}{N}_{i}^{2}/{{{\Sigma }}}_{i}{N}_{i}^{2}$, where *R*_*gi*_ is the radius of gyration for cluster size *N*_*i*_^44^, and find that *ξ* diverges as $\xi \sim {({Z}_{\rm{c}}-\langle Z\rangle )}^{-\nu }$, with *ν* = 0.8 ± 0.1, as shown in Fig. 4b. Finally, we determine the degree of polymerization as the total number of bonded particles. It diverges as ${p}_{m}\sim {({Z}_{\rm{c}}-\langle Z\rangle )}^{-\gamma }$, with *γ* = 0.8 ± 0.1, as shown in Fig. 4c. We thus find three independent critical exponents that coincide well with predictions from percolation models, indicating that our system can be well described as undergoing a percolation-driven, continuous phase transition to the nonequilibrium gel state. Interestingly, the extracted power-law exponents are indistinguishable within error bars from those determined for aggregating weakly attractive colloidal particles^42^, indicating some possible universality of this nonequilibrium percolation process, while the critical coordination number observed there was much higher (*Z*_c_ ~ 5.5), indicating more compact cluster structures. The lower coordination number here indicating more ramified structures is likely due to the much higher attractions between the protein particles, as is reflected in the overall low coordination numbers and prevalence of single-particle strands as shown in Fig. 1.

Independent evidence comes from the emergence of the elastic modulus determined by microrheology, see Fig. 4d. Plotting ${G}^{\prime}$ as a function of mean coordination number, we observe a divergence at a critical coordination number *Z*_c_ = 3.4 ± 0.1, in good agreement with the structural divergence. The data indicate a power-law increase with slope ~1 ± 0.1 (see inset), a value close to the power-law exponent 1 observed in simulated random networks dominated by bond-bending interactions^45^. This correspondence highlights the role of bending interactions in the emergence of rigidity of these slender protein microparticle structures. Together with the irreversible bonding of the protein particles, we hence conclude that the protein microparticle gelation is due to a nonequilibrium percolation process, and the similarity of power-law exponents with previously observed gelation of weakly attractive particles suggests some universality, while the more ramified structure and lower coordination number reflects the stronger interactions of these protein particles.

The critical state of the system close to percolation is further reflected in the size distribution of clusters approaching a power law, as shown in Fig. 4e. In the early stages far away from gelation, this distribution shows an exponential cutoff; as clusters grow, the cutoff moves to the right, until close to gelation, a power law emerges over the full range of cluster sizes, *P* ~ *n*^−*β*^, with exponent *β* ~ 3/2. During this growth process, the clusters exhibit a self-similar structure with a constant fractal dimension, as shown by plotting the size of the clusters as a function of their radius of gyration for different stages of aggregation in Fig. 4f. The constant slope reveals the consistent fractal dimension *d*_f_ = 2 of the aggregating clusters upon approaching gelation. This is in agreement with the fractal dimension of *d*_f_ ~ 2 observed in the secondary aggregation stage of whey protein isolate (WPI) with salt (NaCl) or pH close to the isoelectric point^4,11^. The constant fractal dimension indicates that in the early stages of aggregation, structures form that are of similar, scale-free geometries as those occurring at later stages. Note that for the smallest clusters of ~2 particles, the data deviate as a fractal structure cannot be defined. Following the hyperscaling relation of equilibrium percolation, *d*_f_ relates the scaling of the size of the largest cluster with that of the correlation length via *α* = *νd*_f_. Indeed, we find that even in our nonequilibrium case, the measured exponents *α* = 1.6 and *ν* = 0.8 are in excellent agreement with this relation.

## Discussion

The direct observation of protein microparticle aggregation reveals an intriguing mechanism behind their gelation process: both mechanical rigidity and structure evolution suggest that the protein microparticle gelation is related to a nonequilibrium continuous phase transition with the average coordination number playing the role of an order parameter of the transition, qualitatively in line with recent simulations reporting diverging length scales in the clustering of attractive particles^46^ and with recent studies of the gelation of weakly attractive particles^42^. The relation between the elastic modulus and the mean coordination number suggests that the protein microparticle network is dominated by bending interactions, consistent with the high bending rigidity due to the strong bonding. The universality associated with continuous phase transitions suggests that the mechanism is general and applies to the gelation of many short-range attractive systems, both colloidal and molecular. Indeed, very similar power-law exponents have been observed in the gelation of weakly attractive colloidal particles, albeit with a significantly higher critical coordination number indicating the more compact structure associated with the weaker bonds. Finally, as many proteins are known to form spherical protein clusters before their actual gelation, we hypothesize that our results are of relevance for those proteins as well, pointing out new directions in the understanding of protein gelation. Further experiments and large-scale simulations are needed to consolidate this point.

## Methods

### Experimental details

We make whey protein isolate microparticles by microemulsification of aqueous protein solution in oil with a hydrophobic surfactant, subsequently heated to gel the dispersed proteins within the droplets^19^, as further detailed below and illustrated in Supplementary Fig. 1. The mild heating also leads to slight denaturation of the proteins and exposure of sticky groups. This procedure results in small protein microgel particles that aggregate close to the isoelectric point, i.e., in mildly acidic solution with pH between 4.7 and 5.2 at room temperature (cold-set gelation), allowing us to separate the mild denaturation and preaggregation from the subsequent gelation step^10^. The resulting microparticles have also a well-defined spherical shape and size, and are further size selected by centrifugation, resulting in a particle suspension with a polydispersity of 15% and average diameter 2*r*_0_ = 1.9 μm. The particles are fluorescently labeled with rhodamine to make them visible under fluorescent imaging, see Supplementary Fig. 2. We add 60% by weight sucrose to the aqueous solvent to match its refractive index and density to that of the particles, allowing observation of aggregation deep in the bulk. The density matching prevents particle sedimentation and minimizes any gravitational effects. The added sucrose also slows down the particle diffusion, allowing us to follow the aggregation process in detail. The microparticle diffusion time is *τ* ~ 60 s, in which the particle diffuses its radius. The final suspension has a particle volume fraction of 6%; experiments have been carried out for volume fractions in the range of 3–6% with no qualitative difference in the results. To induce aggregation, we add 0.36% by weight of glucono-*δ*-lactone (GDL), inducing slow acidification and destabilization of the protein suspension (see Supplementary Note 1): the decreasing pH reduces the surface potential of the protein microparticles, approaching their isoelectric point pH = 4.7 after 370 min. Similar behavior is observed for the isolated proteins; their surface potential shows an almost identical dependence on pH with the same isoelectric point, highlighting the close relation of protein and protein microparticle potential, see Supplementary Fig. 3. The resulting gelation process is then followed at constant temperature *T* = 20 °C.

### Materials

To make the protein microparticles, WPI (BiPro JE 192-1-420) with a protein content of 96.4 ± 0.3%, as measured by DUMAS, was obtained from Davisco Foods International Inc. (Le Sueur, USA). The dry matter content of the WPI is 98.6 ± 0.1%. The composition of the whey protein is ~50 wt% *β*-lactoglobulin, ~20 wt% *α*-lactalbumin, and ~30 wt% of a variable mixture comprising bovine serum albumin (~5 wt%), immunoglobulin G (~10 wt%), and proteose peptones (~15 wt%). Polyglycerol polyricinoleate (Grindsted PGPR 90, Denmark), consisting of poly-condensed ricinoleic acid (E476) and the antioxidants alpha-tocopherol (E307) and citric acid, as stated by the manufacturer, was purchased from Danisco (Copenhagen, Denmark). Rhodamine B and GDL were obtained from Sigma-Aldrich (Steinheim, Germany). Acetone (acetone p., CL Chem-Lab), consisting of >99% C_3_H_6_O was purchased from Chem-Lab NV (Zedelgem, Belgium). Sunflower oil (Reddy, NV Vandemoortele, Breda, The Netherlands) and sucrose (van Gilse, Suiker Unie, Oud Gastel, The Netherlands) were bought at a local supermarket and used without further purification. Throughout all experiments, demineralized water was used, which was purified using a Milli-Q apparatus (Purelab Ultra, ELGA, High Wycombe, UK).

### Protein microparticle synthesis

Cold-set gelation in general is a method that requires a dispersion of “activated” proteins or protein particles, which tend to aggregate and form a network upon a change in the conditions of the system. As the functionality of proteins is not only related to their intrinsic physical and chemical properties but also to their conformational properties^47^, denaturation, i.e., a change in the native conformation, is commonly the prerequisite to “activate” the proteins. Consequently, cold-set gelation is a two-step process^48^, which provides the unique feature to uncouple the denaturation of the proteins from the subsequent steps in the gelation process. The first step of cold-set gelation includes a heat treatment of a solution containing native proteins at neutral pH and low ionic strength, at a concentration below their critical gelling concentration. To induce conformational changes, the heating temperature should be above the denaturation temperature, which is typically 70–90 °C. This results in the formation of small protein aggregates, which remain dispersed upon cooling down the obtained solution to ambient temperature. Subsequently, acid-induced gelation is achieved by adjusting the pH towards the isoelectric point of the proteins. This causes a reduction in the electrostatic repulsion between the protein aggregates, thereby enabling the formation of a space-spanning gel network. Thus acid-induced gelation differs substantially from heat-induced gelation. In heat-induced gelation, the charge density of the proteins remains unchanged throughout the entire process, while the increased hydrophobic and hydrogen interactions cause aggregation of the proteins.

We prepared heat-denatured WPI microbeads by modifying the double-emulsification method described by Saglam et al.^19^. An aqueous stock solution containing 25% (w/w) WPI was prepared by dissolving the respective amount of protein in demineralized water and stirring for 2 h, followed by overnight storage at 4 °C to allow for full hydration of the proteins. In addition, an oil stock solution was prepared by dissolving 5% (w/w) PGPR in sunflower oil by stirring for at least 2 h. The subsequent formation of microparticles is illustrated in Supplementary Fig. 1. To form small droplets of WPI solution, the WPI stock solution was added dropwise at a speed of 1 mL/10 s to the oil stock solution up to a weight fraction of 20%, while being mixed using a rotor-stator homogenizer at 5000 r.p.m. (Ultra-turrax T 25, IKA Werke, Germany). After the complete addition of the WPI stock solution, the sample was mixed for an additional 5 min while being kept on ice to prevent it from overheating. The obtained w/o emulsion, composed of WPI stock solution droplets dispersed in the oil stock solution, was subsequently heated in a temperature-controlled water bath at 80 °C for 20 min. This heating step transformed the WPI solution droplets into heat-induced WPI microgel beads. The microbead-in-oil suspension was subsequently cooled on ice to room temperature and centrifuged (Avanti J-26 XP, Beckman Coulter, U.S.A) at 36,000 × *g* for 1 h to remove the majority of the oil. The pellet, which was mainly composed of the WPI microbeads, was then redispersed in acetone at a ratio of 1:10 (w/w) and stirred overnight to allow the acetone to extract the oil and the water from the microbeads. Each 50 mL of the resulting suspension was subsequently filtered on a cellulose filter (WhatmanTM 5, diameter 90 mm) and rinsed with 25 mL clean acetone. Finally, the retentate containing the washed WPI microbeads was dried overnight under continuous air flow at room temperature to allow the residual acetone to evaporate, yielding a dry flaky powder composed of WPI microbeads. The dried microbeads were gently removed from the filter and stored in a glass container at room temperature until further use.

### Measurement of the pH evolution

To obtain an indication of the development of the pH during the aggregation process, 2 mL of the final sample of protein microparticles in the sucrose aqueous solution were transferred into a 4 mL glass vial at 25°C and the pH was recorded every 30 s over a time interval of 700 min using a pH-stat system (Metrohm, Herisau, Switzerland). The same measurement was done on the regular protein suspension. To be most representative, these pH measurements were always performed on the same day as the aggregation measurements.

### Confocal microscopy and particle tracking

We use a laser-scanning confocal microscope (Zeiss 5 Live) to image the fluorescently labeled protein microparticles in three dimensions. Stacks of 200 images with a vertical separation of 0.2 μm between images were recorded every 5 min. In addition, a time series of 5000 single images were recorded at a frame rate of 24 s^−1^ and a fixed height of 20 μm from the bottom of the sample cell, avoiding any influence of the boundary. Typical images of the image stacks are shown in Supplementary Fig. 2. These show the aggregating protein solution at an early (dispersed), intermediate (clustered), and late (gelled) state. We use the open-source particle tracking software trackpy, python-based code of particle tracking software originally written in IDL. The software provides an uncertainty estimation, based on the signal-to-noise ratio of the images and the radius of gyration of the features, following the relation

1 $$\epsilon =\frac{N}{S}\frac{{l}_{\rm{noise}}}{2{\pi }^{1/2}}\frac{{w}^{2}}{{a}^{2}}$$

where *l*_noise_ is the noise correlation in pixel length, *N*/*S* is the noise to signal, and *w* and *a* are the feature size and mask size, respectively. Based on the standard deviation of the error that follows from this approach, we estimate a horizontal locating accuracy better than 20 nm, and vertical locating accuracy better than 35 nm.

## Supplementary information

Supplementary Information

## Data Availability

The datasets generated during and/or analyzed during the current study are available from the corresponding author on reasonable request.

## Code availability

The codes used to analyze the data are available from the corresponding author on reasonable request.
