# Supplementary material for: Protein microparticles visualize the contact network and rigidity onset in the gelation of model proteins
Source: NPJ Sci Food. 2021 Dec 13;5:32. doi: 10.1038/s41538-021-00111-5 (PMC8668889; doi:10.1038/s41538-021-00111-5)
Supplement: Supplementary file 1 — Supplementary Information [file 41538_2021_111_MOESM1_ESM.pdf]

Supplementary Information for article
”Protein microparticles visualize the contact network and rigidity onset in the gelation of model proteins”

Rouwhorst *et al.*

SUPPLEMENTARY NOTE 1: INTERACTION POTENTIAL

The gradual decrease in pH due to the addition of glucono-δ-lactone brings the proteins closer to their isoelectric point. As a result, the electrostatic repulsion between the particles diminishes, and the attractive van-der-Waals and hydrophobic interaction forces dominate, leading to aggregation. We estimate the underlying protein-protein interaction potential in the framework of DLVO theory. The electrostatic repulsion is computed from the measured zeta potential ζ according to

$$V_{\mathrm{el}} = 2\pi r \zeta^2 / \kappa^2 \epsilon_0 \epsilon * e^{-\kappa h}, \tag{1}$$

where the Debye screening length κ^{-1} is estimated from the ionic strength I_{ion} at a given pH taking into account the added 5mM KCL using

$$\kappa^{-1} = \sqrt{\frac{\epsilon_0 \epsilon_d k_B}{2 I_{ion} N_A}} \tag{2}$$

The van-der-Waals attraction is computed from the Hamaker constant H and the particle center-to-center distance h according to

$$V_{\mathrm{vdW}} = \frac{r * H}{12 * h}, \tag{3}$$

where we take $r = 1.1\mu m$, the average particle radius, and the Hamaker constant $H = 3k_BT$, as found for similar protein interactions in water [4–6]; we thus assume the same Hamaker constant for the microparticles as for the continuous protein phase.

The resulting total potential

$$V_{tot} = V_{\mathrm{el}} - V_{\mathrm{vdW}}. \tag{4}$$

exhibits a change from strongly attractive to repulsive at a Zeta potential larger than $\sim 13mV$, see Supplementary Figure 3a. To link this estimated potential back to the experimental measurements of aggregation, we plot the evolution of pH as a function of time after adding glucono-δ-lactone in Supplementary Figure 3b. We also measured the Zeta potential of the WPI microbeads as a function of pH by electrophoresis, as shown in Supplementary Figure 3c. For these measurements, the WPI microbeads were dispersed in demineralized water at a concentration of 0.1% (w/w), and the Zeta potential was measured at 7 different pH values ranging from 3.0 to 7.0 using a Nano ZS Zetasizer (Malvern Instruments). Supplementary Figures 3b and c together allow us to estimate the time evolution of the microparticle potential and compare it to the time scale of aggregation. We see from Supplementary

Figure 3a that the repulsive barrier in the DLVO potential has completely disappeared at a Zeta potential of 13mV; according to Supplementary Figure 3c, this happens at a pH of about 5.2, which, according to Supplementary Figure 3b, corresponds to a time of 225 minutes. This time indeed corresponds to the onset of aggregation, as shown in Supplementary Figure 3d. Thereafter, the pH evolves only slowly, leading to even higher attraction, which however is not so relevant any more for the aggregation behavior, as already for the Zeta potential of 13mV the estimated interaction energy is $-30k_BT$, causing irreversible particle attachment with very low probability of rearrangement. The gelation process thus proceeds by irreversible aggregation, greatly slowed down by the increased viscosity due to the high concentration of sucrose in the solution. We note that the sugar can influence the protein interactions: the lower dielectric constant can slow down the atomic motion in the protein molecules [7], and the added sugar decreases the Hamaker constant, reducing the Van der Waals interaction, which could further slow down the aggregation process. The value found for the isoelectric point of the WPI microbeads is in good agreement with the values ranging from 4.7 to 5.0 reported by other authors for WPI aggregates produced in aqueous solutions at neutral pH [8, 9]. Moreover, the value is in perfect agreement with the isoelectric point of heat-induced whey protein aggregates produced by simply heating the same specific protein isolate in an aqueous solution below its critical gel concentration, as shown in Supplementary Figure 3c. Therefore, the microparticles mimick the expected isoelectric properties of the heat-denatured WPI aggregates.

SUPPLEMENTARY NOTE 2: MICRORHEOLOGY

Microrheology relates the recorded displacements of features in soft samples to the local visco-elastic behaviour of the material. This non-invasive method gives insight into the local mechanical response of a wide range of soft materials, including colloidal gels and living cells. Several software distributions have been developed for microrheology, including the software package of J. Crocker for the interactive data language (IDL), which was translated and expanded upon in Matlab [1]. We use an algorithm based on a python adaptation of this software [2]. The tracer particles' mean square displacement, $< \Delta r^2(\tau) >$, is related to

the creep compliance, J, according to [3]:

$$J(\tau) = \frac{3\prod a}{N_d k_B T} < \Delta r^2(\tau) >, \tag{5}$$

where a is the average particle radius N_d is the dimensionality of the motionk_B the Boltzmann constant and T the temperature. The complex moduli are related to the creep compliance via:

$$G^*(\omega) = \frac{1}{i\omega\widetilde{J}} \tag{6}$$

We track the individual protein particles and determine their mean-square displacements over 100 seconds from a set of 2000 images taken at a frame rate of 20^{-1}. Using these particles of the protein network themselves as tracer particles and combining the result of a large quantity of particles ($\sim$ 400), we obtain the complex moduli over time shown in Fig. 2 of the main text. The storage modulus overtakes the loss modulus as the system approaches a gel state, increasing roughly an order of magnitude compared to the early stages of aggregation. This can be seen in more detail in the full frequency-dependant moduli taken at different stages of aggregation in Supplementary Figure 4. At the start of aggregation, the fluid state is characterized by a power-law storage modulus ($\propto \omega^1$). As aggregation proceeds and gelation is approached, the modulus increases and flattens towards low frequencies, reflecting the emergence of solid-like behaviour of the system upon gelation.

Compared to the storage modulus, the loss modulus at the start of aggregation shows higher values over most of the frequency range (Supplementary Figure 4). As time evolves, the moduli increase in magnitude, and the storage modulus overtakes the loss modulus over a wide frequency range, in particular at low frequencies.

SUPPLEMENTARY REFERENCES

[1] M. Kilfoil, Matlab algorithms from the Kilfoil lab., `http://people.umass.edu/kilfoil/downloads.html`

[2] Maier, T., Haraszti, T., Python algorithms in particle tracking microrheology. Chem Cent J., 6:144 (2012).

[3] Mason TG, Weitz D.A. Optical measurements of frequency-dependent linear viscoelastic moduli of complex fluids, Phys. Rev. Lett. 74 (1995) 1250 – 1253.

[4] Lund, M. and Jönsson, B. A mesoscopic model for protein-protein interactions in solution. Biophys. J. 85, 2940–2947 (2003)

[5] Mezzenga, R. and Fischer, P. The self-assembly, aggregation and phase transitions of food protein systems in one, two and three dimensions. Reports Prog. Phys. 76, 046601 (2013).

[6] Asthagiri, D., Nea;, B, B. L.and Lenhoff, A. M. Calculation of short-range interactions between proteins. Biophys. Chem. 78, 219-231 (1999).

[7] R. Affleck, C.A. Haynes, and D.S. Clark, Proc. Nat. Acad. Sci. 89, 5167-5170 (1992).

[8] Jean K., Renan, M., Famelart, . H., Guyomarc'h, F. Structure and surface properties of the serum heat-induced protein aggregates. Int. Diary J. 16, 303-315 (2006).

[9] Morand, M., Guyomarc'h, F., Legland, D., Famelart, M. H., Changing the isoelectric point of the heat-induced whey protein complexes affects the acid gelation of skim milk. Int. Diary J. 23,9-17 (2012).

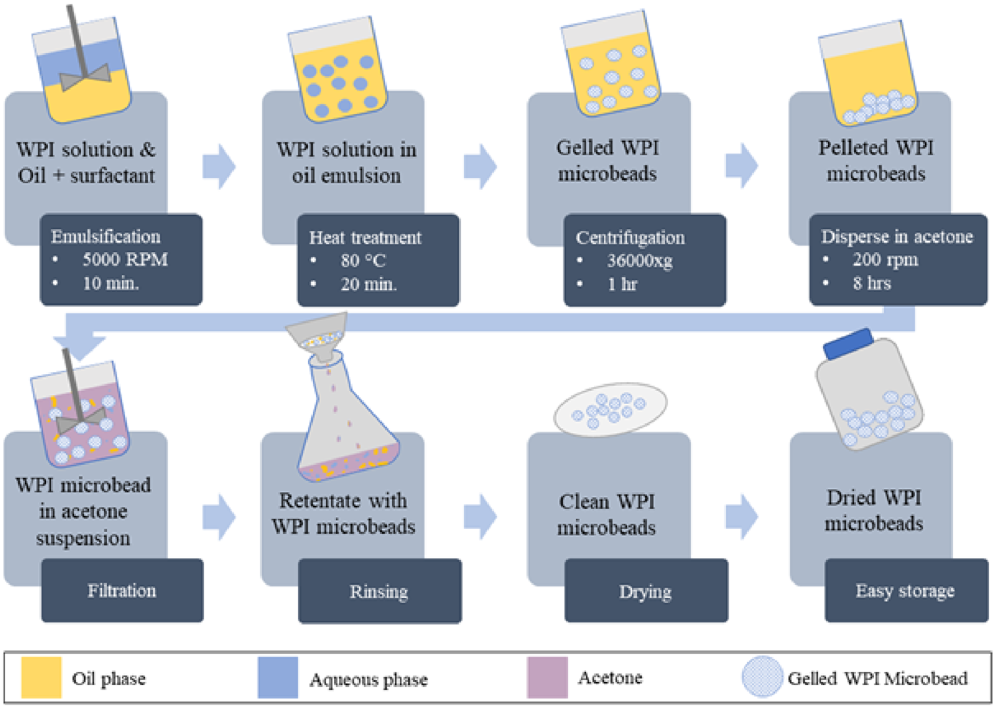

Supplementary Figure 1. **Schematic overview of the preparation procedure of dry WPI microbeads**

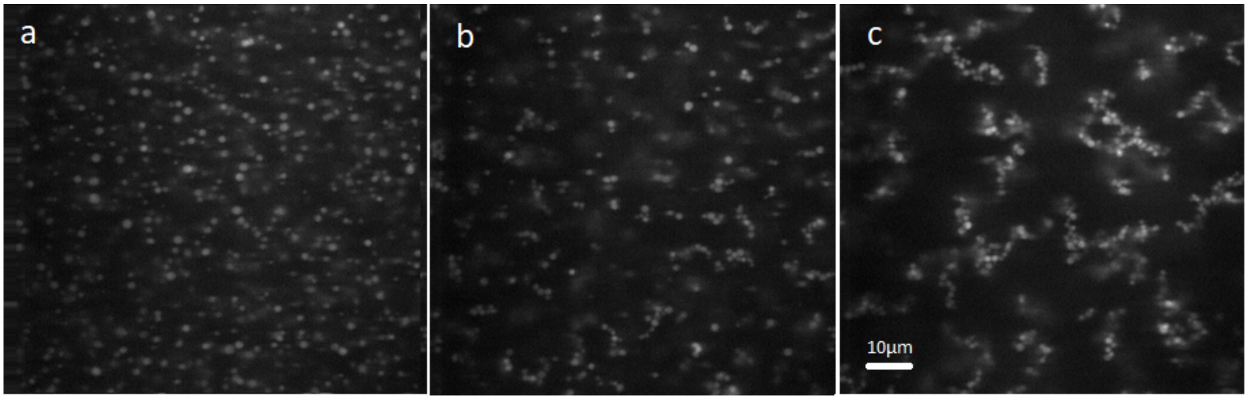

Supplementary Figure 2. **Confocal microscope images of the protein microparticles.** Images show examples of the dispersed (a) , cluster (b) and gelled (c) phases.

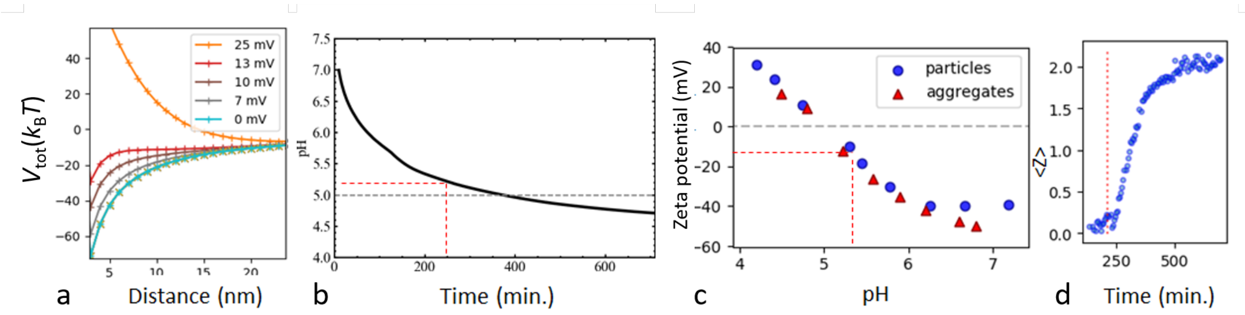

Supplementary Figure 3. **Interaction potential.** (a) DLVO potentials for various zeta-potentials reveal the electrostatic barrier disappears when the zeta potential approaches a value of 13mV. (b) Measurement of the pH of the acidifying solution as a function of time. The data shows we approach a pH of 5.2 after 225 minutes. (c) Zeta potential as a function of pH measured by electrophoresis. The zeta potential corresponding to pH 5.2 is 13mV. The isoelectric point is found to be at the same pH for both the WPI microparticles (blue dots) as well as for the protein aggregates (red triangles), reflecting identical electrostatic properties for WPI and WPI microparticles. (d) Mean coordination number of the particles as a function of time reveals that pH 5.2 is reached in the very early stage of the aggregation process (vertical red dotted line).

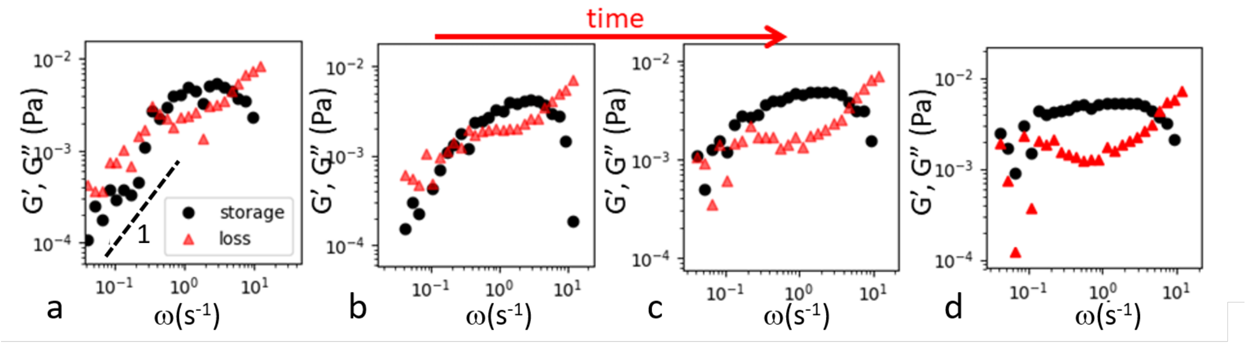

Supplementary Figure 4. **Evolution of the frequency-dependent moduli.** The frequency-dependent storage and loss moduli determined from microrheology show the transition from a fluid to a solid material from the rise of the low-frequency response. Dashed line in (a) indicates power-law slope 1. At the start of aggregation (a), the storage modulus shows lower values than the loss modulus at low frequency. As time evolves, the moduli increase in magnitude, and the storage modulus overtakes the loss modulus over a wide frequency range (d).
